# Agentic appeals increase charitable giving in an affluent sample of donors

**DOI:** 10.1371/journal.pone.0208392

**Published:** 2018-12-06

**Authors:** Ashley V. Whillans, Elizabeth W. Dunn

**Affiliations:** 1 Negotiations, Organizations, Markets Unit, Harvard Business School, Harvard University, Cambridge, Massachusetts, United States of America; 2 Department of Psychology, University of British Columbia, Vancouver, British Columbia, Canada; University of Toronto, Rotman School, CANADA

## Abstract

Recent research suggests that affluent individuals adopt agentic self-concepts, striving to stand out from others and to master the environment on their own. The present study provides a road test of this idea, showing that this theorizing can be utilized to increase charitable giving among the affluent, when individuals do not realize that their behavior is being studied. In a naturalistic field experiment conducted as part of an annual fundraising campaign (*N* = 12,316), we randomly assigned individuals from an affluent sample to view messages focused on agency (vs. communion). Messages that focused on personal agency (vs. communion) increased the total amount of money that individuals in the sample donated by approximately 82%. These findings provide evidence for a simple, theoretically-grounded method of encouraging donations among those with the greatest capacity to give.

## Introduction

It might seem obvious: wealthier individuals should be the most financially generous. After all, wealthy individuals are in the best position to help those in need. Yet, some studies find that wealthier individuals donate a smaller proportion of their income to charity each year as compared to less affluent individuals [1,2]. Other studies, however, suggest that there is no relationship [3] or even a positive relationship between affluence and financial generosity [4]. In trying to make sense of these findings, we argue that it is worth considering the match—or mismatch—between the mindset associated with wealth and typical messages about charitable giving.

Previous research suggests that individual differences in wealth are associated with differences in self-concepts (i.e., how people think about themselves). For example, wealthier individuals typically develop more agentic self-concepts, defining themselves primarily through their own capacity for personal control [5]. As a result, wealthier individuals report higher perceptions of control over daily events and show a greater desire to make decisions for themselves [6]. In essence, money enables people to achieve their goals without help from others, and thus, wealthier individuals typically adopt agentic self-concepts, striving to stand out and master their environments on their own [5,6].

It is important to note that these motivations to enact personal control and to prioritize personal success typically stand in conflict with the motivation to value one’s community and to help others [7,8]. Research in social psychology and behavioral economics suggest that messages are more impactful when they fit people's underlying motivations [9, 10]. However, charitable giving is typically framed as a communal activity that involves joining together to benefit others [11–13], which does not align with the agentic self-concepts commonly found among wealthy individuals. Building on this research, we investigated the effects of flipping the framing of charitable giving to fit with the agentic goals and motivations that are often embraced by the wealthy.

Our research question follows from a great deal of psychological research showing that when messages fit underlying motivations, people are more likely to engage in an intended behavior (such as charitable giving). In economics, there are also several models that seek to predict when people will give to charity. For example, the warm-glow model posits that charitable giving is supply-driven and that it is utility maximizing for the giver to give [14,15]. In contrast, the social pressure model [16] posits that charitable giving is demand driven, and that giving is utility-reducing for the giver. While it is not our intention to speak to these economic models of charitable giving in the current paper, it is possible that agentic messages could increase charitable giving by helping affluent individuals recognize the potential personal benefit of the giving opportunity. This rationale would be consistent with the ‘warm-glow’ model of giving. In fact, our data is consistent with this theorizing because donors in our study made their donations privately and therefore experienced little to no social pressure to donate in the context of this naturalistic experiment [see 17 for a similar argument].

Following from our own and others’ research, we reasoned that wealthier individuals should respond more positively to charitable appeals that emphasize agency (the pursuit of personal goals) as compared to charitable appeals that emphasize communion (the pursuit of shared goals). To provide an initial test of this idea, in a recently published paper, we conducted three experiments with working adults from a diverse range of socioeconomic backgrounds [18]. In each study, we measured participants’ annual household income and examined how the wording of solicitations influenced charitable donation decisions. Across these studies, we found that messages focusing on agency (vs. communion) increased charitable donations for individuals with household incomes above $90,000 USD.

While informative, this research has two features that limit our ability to assume that our messaging strategy could be successfully applied in the real world. First, our research relied on the use of controlled studies, where participants knew that their responses were being recorded. Although we allowed them to make their donation decisions in private, people often act differently when they know that their behavior is being studied [19]. Second, our approach entailed asking participants to report their household income, which is sensitive, private information that may often be unavailable to organizations soliciting donations.

To overcome these limitations, we conducted a naturalistic field experiment with over 12,000 respondents during an annual fundraising campaign. Importantly, potential donors did not know that their responses were being recorded as part of a study. Rather than asking donors to report their own wealth, we used an indirect indicator that is readily available to organizations: the median household income in respondents’ zip code. The current study therefore enables an externally valid field test of the idea that agentic (vs. communal) appeals could encourage charitable giving among the affluent.

The current study was conducted in parallel with two independent investigations examining the impact of donor choice on the charitable giving behavior of alumni. In one field experiment [20], researchers randomly assigned 32,174 alumni of an Ivy League university in the US to receive one of two mail-outs. Alumni either received a standard mailer or a mailer that allowed participants to choose one of the four priorities of the university’s fundraising campaign that was most important to them. In this experiment, the richest five percent of alumni donated $12.07 more on average when they were assigned to the “choice” vs. control condition. In another field experiment [21], researchers randomly assigned 10,600 alumni of a large public research university in the US to receive one of two mail-outs. Alumni either received a standard mailer or a mailer that allowed them to direct their giving to one of two university fundraising campaigns. In this experiment, alumni who were able to direct their giving donated $7.61 more on average than alumni who received the standard mailer. Together, these field experiments provide evidence that appeals that provide donors with a sense of agency by presenting choice over elements of the donation decision can increase charitable giving. The first experiment also suggests that this approach may be the most effective among those with the greatest capacity to give.

Of course, it is not always possible to provide donors with a feeling of choice over the outcome of their donations. Thus, in the current study, we examined whether simply changing the language of the appeal might also be effective at encouraging charitable giving. Specifically, we manipulated the language of the appeals used during an annual fundraising campaign at an elite university in the US, and examined whether agentic (vs. communal) messages increased charitable giving.

Following from [20], it is possible that agentic (vs. communal) messages would increase charitable giving only among the wealthiest donors in our study. We therefore might expect to observe an interaction between message frame and wealth to predict donation amount. However, [18] find that agentic (vs. communal) messages are more effective for respondents with incomes above $90,000 USD. Because we conducted our experiment with a large sample of wealthy alumni from an elite business school, it is possible that we might observe a main effect of the agentic (vs. communal) message on donation amount. In light of these two competing possibilities, we examined whether the agentic (vs. communal) message had an overall positive effect on the amount donated to the campaign and whether the agentic (vs. communal) message was only effective among the wealthiest participants in our sample. Consistent with the first possibility, agentic (vs. communal) messages had an overall positive effect on amount donated in this study. We now describe the study methods and results in more detail.

## Materials and methods

### Participants & procedure

We tested the impact of agentic (vs. communal) appeals during an annual fundraising campaign at an elite business school in the US (*N* = 12,316). This campaign took place during the month of December 2015. During the campaign, potential donors—all of whom were graduates of the business school—received two emails and a letter prompting them to donate. We randomized individuals on the mailing list to receive messages that focused on agency or communion. Participants were not aware that they were in a study, informed consent was waived by the ethics committee. The study was approved through the Human Ethics Boards at the University of British Columbia and Chicago University.

All participants received both the tailored emails and the mail-out, and all materials contained the identical manipulation. The fundraising office tracked donations in response to the letter and emails. We totaled participants’ donations to the letter and emails to create an overall index of campaign donations. We report donations to each separate communication in the Supplemental Material (see S1 Text). Importantly for this research, our sample was relatively wealthy (See Table 1 for the detailed demographic characteristics of this sample). According to a report issued by the partner university in 2015 (the year that we conducted this study), graduates of this business school earned an average *starting* salary of $100,000/year. To provide context, in 2015, the average starting salary for graduates of bachelor degree programs in the US was $45,000/year [21]. Using 2014 US Census data, we can estimate that the average donor in this study lived in a neighborhood with a median household income of $85,160.00 USD. Random assignment was successful: there were no significant differences between respondents who were randomly assigned to the agentic condition or the communal condition (Table 2).

**Table 1:** Participant characteristics.

	% Donated	*M*. Donation Amount (Sample Donated)	*M*. Donation Amount (Full Sample)	% Female	% Caucasian	Age (Range)	Income (Range)
	4.1%	$524.54 ($2,428.27)^a^	$21.54 ($502.51)	23.4%	80%	53.0 (28–103)	$85,160.00 ($12,076 to $245,600)^b^
***N***	12,316	500	12,316	12,316	8,487	11,249	12,134

^a^The value reported represents the average donation amount contingent on donating.

^b^This value was extrapolated from the 2014 US Census and represents the median income in the zip code each donor reported living in

**Table 2:** Demographic characteristics by condition.

	% Female	% Caucasian	Age	Income	% Warm List Donors in 2015	% Warm List Donors (Ever)	2015 donation amount	# of Yrs of Consecutive Giving	Years Since Last Gift
**Agentic**	23.2%	79.4%	53.89 (14.15)	$89,930.17 ($34,644.81)	26.8%	67.1%	$149.80 ($994.44)	1.48 (4.62)	8.23 (9.08)
**Communal**	23.6%	80.6%	54.01 (13.95)	$90,431.42 ($34,920.09)	26.2%	66.5%	$131.50 ($681.93)	1.42 (4.30)	8.48 (9.17)
**p-values**	0.575	0.171	0.650	0.427	0.474	0.502	0.233	0.476	0.145

#### Message frames

The messages that we tested in this study were adapted from solicitations that the university was already using. See Table 3 for the messages participants received during the campaign in each condition. Before conducting our field study, we tested these messages with *N* = 52 MBAs recruited from the same participant pool to ensure that the messages differed only on agency and communion and not on other relevant characteristics. Our pilot test confirmed this assumption (Table 4).

**Table 3:** Wording of messages.

	Message at the top of the solicitation	Message at the bottom of the solicitation
**Communal**	“Sometimes, one community needs to come forward and support a common goal. This is one of those times.”	“Join your community and support a common goal. Donate today.”
**Agentic**	“Sometimes, one person needs to come forward and take individual action. This is one of those times.”	“Come forward and take individual action. Donate today.”

**Table 4:** Table of means and standard deviations for the pilot test results (N = 52).

Item	“To what extent does this appeal emphasize agency?”	“To what extent does this appeal emphasize communion?”	“I think contributing to this cause would increase my social status.”	“I think contributing to this cause would make me feel more powerful.”	“I think contributing to this cause would make me feel like a more important person.”
**Agentic *M*(SD)**	4.27 (1.61)	4.17 (1.65)	3.46 (1.50)	3.94 (1.60)	4.17 (1.62)
**Communal *M*(SD)**	3.65 (1.49)	4.88 (1.20)	3.38 (1.39)	3.88 (1.57)	4.13 (1.58)
**Statistics**	*t*(51) = 2.80, *p* = 0.007	*t*(51) = 3.85, *p* < 0.001	*t*(51) = 1.07, *p* = 0.289	*t*(51) = 0.69, *p* = 0.497	*t*(51) = 0.28, *p* = 0.785

Participants were asked to rate their agreement with each item on a scale from (*1* = Strongly Disagree to *7 =* Strongly Agree).

## Results

### Overview

We conducted three types of statistical analyses to assess the effect of message type on donation behavior. We first compared whether individuals who viewed the agentic message were more likely to donate than individuals who viewed the communal message using probit regression. We then compared the amount of money that individuals donated between the agentic message group and the communal message group using pairwise mean comparisons. Next, we conducted ordinary least squares regression to ensure that demographic characteristics such as gender, age, and donation history could not explain our results. In this study, 4.1% of respondents donated (*N* = 494). Due to the high number of alumni who did not donate to the campaign, the donation data were not normally distributed. To ensure that the results were robust to various model specifications, we report our results using OLS regression with a raw donation amount outcome variable, OLS regression with a log transformed donation amount outcome variable, and Tobit regressions to account for the large proportion of respondents who did not donate. To compute log transformed analyses without deleting 0’s, we added +1 to all values prior to log transformation. In the Supplemental Material (Table B in S1 Text and Table C in S1 Text), we report additional analyses showing that our results were not driven by the small number of alumni who donated much more than average to the fundraising campaign (by winsorizing donations at the 99^th^, 95^th^, and 90^th^ percentiles). The main findings of this study were consistent across various model specifications and analytic strategies: Messages focused on agency (vs. communion) increased the total amount of money that respondents donated to the campaign.

### Donation likelihood

After viewing the agentic message, alumni were no more likely to donate during the campaign than after viewing the communal message, *B =* 0.06 (0.04), *X*^*2*^(1, 12,315) = 1.97, *p* = 0.160, *η*^2^ = 0.0001. These results held controlling for critical covariates including participants’ age, gender, whether participants had donated to the annual fund campaign in 2015, whether participants had ever donated to the campaign, and the consecutive years that participants donated. See Supplemental Material for the full regression models for these analyses (Table A in S1 Text).

### Donation amount

After viewing the agentic messages, alumni contributed an average of $18.47 USD. After viewing the communal messages, alumni contributed an average of $10.13 USD. This $8.34 difference was statistically significant, *F*(1, 12,315) = 9.31, *p* = 0.001, *η*^2^ = 0.001. These statistically significant results held when we used the log-transformed donation variable as our key outcome of interest, *F*(1, 12,315) = 3.90, *p* = 0.048, *η*^2^ = 0.001. These statistically significant results also held controlling for covariates that could have otherwise explained these findings including participants’ age, gender, whether participants had donated to the annual fund campaign in 2015, whether participants had ever donated to the campaign, and the consecutive years that participants donated. See Supplemental Material for additional analyses (Table B in S1 Text).

Looking only at people who donated to the campaign (*N* = 494), agentic messages increased the amount of money that people donated. After viewing the agentic messages, participants who contributed to the campaign donated an average of $431.70 USD. After viewing the communal messages, participants who contributed to the campaign donated an average of $270.30 USD. This $161.40 USD difference was statistically significant, *F*(1, 493) = 7.16, *p* = 0.008, *η*^2^ = 0.014. Once again, these statistically significant results held when predicting log donation amount and when controlling for covariates that could have explained these findings including age, gender, whether participants had donated to the annual fund campaign in 2015, whether participants had ever donated, and the consecutive years that individuals donated. We also conducted Tobit regression analyses. Again, alumni contributed more money when they viewed the agentic vs. communal message. See Table B in S1 Text for additional analyses.

Although we observed an overall effect of the agentic (vs. communal) message on donation amount, recent research suggests that charitable appeals focused on donor agency might be the most effective among wealthier individuals [18]. Building on this research, we conducted additional analyses to examine whether the agentic (vs. communal) messages were especially effective for the wealthiest individuals in our sample. To examine this question, we conducted interaction analyses using a readily available index of respondents’ wealth: the wealth of respondents’ neighborhoods (zip code). In response to a reviewer’s suggestion, following [20], we assessed whether there was an interaction between condition assignment and respondents’ job title to predict donation amount (1 = CEO or 1 = CEO+Board Member). This interaction did not predict donation amount across any of the above model specifications and is not discussed further.

### Wealth

To examine whether the agentic messages were most effective for the wealthiest individuals in this sample, we assessed whether there was an interaction between condition and the wealth of respondents’ zip codes to predict donation amount. In contrast to this hypothesis, the interaction between condition and household income was not significant, *F*(1, 12,312) = 0.08, *p* = 0.775. These results were statistically similar when predicting log donation amount, *F*(1, 12,124) = 1.05, *p* = 0.305. These results suggest that the effectiveness of the agentic (vs. communal) messages did not differ depending on the wealth-level of respondents’ neighborhoods.

### Past donation history

On an exploratory basis, we also examined whether condition assignment interacted with the amount of money that respondents had previously donated to the fundraising campaign. In these analyses, there was a statistically significant interaction between condition assignment and donation history to predict donation amount, *F*(1, 12,312) = 100.93, *p*<0.001. We then used the Johnson-Neyman procedure to examine the levels of income whereby the agentic vs. communal appeals became more effective. This procedure is used to identify the point(s) along a continuous moderator whereby the relationship between the independent variable and the outcome variable transitions from statistically significant to statistically non-significant [22].

These follow up spotlight analyses revealed that the agentic (vs. communal) messages encouraged larger donations for alumni who had donated at least $6,653 USD total to previous university fundraising campaigns (22.01% of the donors in this study). For alumni who had donated less than $6,653 USD in previous fundraising campaigns, the agentic and communal appeals were equally effective. These results were statistically similar when predicting log donation amount, *F*(1, 12, 303) = 7.56, *p* = 0.006. These results held controlling for age, gender, income, whether individuals donated to the campaign in 2015, and years of consecutive donations. See Supplemental Material (Table C in S1 Text). These results suggest that the effectiveness of the agentic (vs. communal) messages differed based on the amount of money that respondents donated to previous campaigns: alumni who had donated more money to previous university fundraising campaigns were more likely to be influenced by the agentic (vs. communal) messages.

## Discussion

This study provides evidence that agentic (vs. communal) messages can increase donations among the affluent in a large-scale, real-world fundraising campaign. As compared to communal messages, messages that framed charitable giving as an agentic act increased donation amounts by an average of $8.34, representing an 82.3% increase. Although the various model specifications point to the fact that the intervention had a statistically small impact on donation amount in this study, given that this result was obtained in the context of a costless intervention, the increase that we observed could be of practical interest to non-profit organizations.

While past research has found evidence that messages that provide donors with agency are especially effective for the wealthiest individuals [18, 20], we found mixed evidence for this idea. Our index of wealth (median household income by respondents’ zip code) did not moderate the results that we observed in this field experiment. It is possible that we were unable to detect an interaction between wealth based on zip code data and condition assignment due to the imprecision that is associated with measuring respondent-level wealth using a measure of the wealth of respondents’ neighborhoods. It is also possible that the majority of respondents were more motivated by agentic messages, since the majority of respondents likely made above the $90,000 USD per year threshold observed in past research [18].

We did find evidence that agentic appeals were particularly motivating for engaged donors. The manipulation of agency that we used in this study emphasized the role of the individual, highlighting the importance of “one person coming forward to take action.” It is therefore possible that the agentic (vs. communal) message was effective because it evoked respondents’ sense of identity as a donor to their university. This explanation is consistent with a field experiment showing that donors who have recently contributed to a cause are more persuaded by identity-related appeals as compared to donors who have not recently contributed to a cause [10]. Together, these findings point to the possibility that the agentic (vs. communal) messages used in this study were effective because they appealed to alumni’s identity as a donor, and not necessarily because these messages provided a better “fit” with the agentic mindsets that are typically associated with having a higher financial position in society.

It is also possible that communal messages suppressed participants’ willingness to give to the campaign as these appeals emphasized the role of other (affluent) people in one’s network engaged in the “common goal” of giving. This argument is consistent with research showing that imagining other people can reduce helping through diffusion of responsibility [23]. Future work should explore these mechanisms.

Indeed, our data cannot clearly disentangle the specific mechanisms by which agentic (vs. communal) messages increased giving. Regardless of the specific mechanisms that underpin these findings, our data point to an actionable conclusion: When trying to increase charitable giving among a relatively affluent sample of individuals, it is likely worth using agentic (vs. communal) appeals. When working with a relatively affluent sample, these data suggest that using agentic messages may encourage giving and at the very least, will not discourage it.

Research suggests that wealthier individuals donate more in response to agentic messages. The current data extends past findings by showing that agentic messages have an overall positive effect on real-world donation decisions in a relatively affluent sample. Together, this combination of tightly controlled experiments and an ecologically valid field experiment provide evidence that agentic (vs. communal) messages increase giving among the relatively affluent.

Although our messaging intervention influenced the size of donations, there was no statistically significant effect of message type on the percentage of people who chose to donate in this study (See Table A in S1 Text). These findings are consistent with the results of other naturalistic field experiments, which have shown that donation amount is often more responsive to framing manipulations than new donor participation rates, in part because people who make donations to annual fundraising campaigns often tend to be regular donors [24–26]. In the current study, 86.3% of respondents who donated to the annual fund campaign had donated to previous campaigns. Thus, more research is needed to examine when agentic messages might also increase new donor participation. More research is also needed to examine whether the benefits of agentic (vs. communal) messages on donation decisions persist over time.

This experiment is a test of the effectiveness of agentic messages in only one context (e.g., among wealthy alumni at an elite business school in the US). It would therefore be worthwhile to test the efficacy of these messages in other giving-related contexts. For example, although we only examined financial generosity, portraying generosity as an opportunity to satisfy agentic vs. communal goals may be differentially effective at promoting a variety of prosocial behaviors among relatively affluent individuals—from donating blood to volunteering at soup kitchens. Agentic messages might also be more effective at increasing generosity in contexts where the overall mission of the organization focuses on agency (e.g., a charity that funds entrepreneurs) as compared to contexts where the overall mission of the organization focuses more on communal goals (e.g., a charity that helps children) or where the organization is perceived as more effective (vs. less effective). Furthermore, eliminating the motivational conflict between wealth and generosity should not only promote higher levels of prosocial behavior, but may also increase the satisfaction that individuals gain from helping other people. Depending on how these initial acts of generosity are framed, these initial prosocial acts may be more self-reinforcing and therefore produce more sustainable increases in helping.

Broadly, this investigation is relevant to an important set of questions in psychology and economics: how can we encourage individuals to engage in behaviors that involve personal sacrifice for the benefit of society? [27] This investigation also answers a call for social scientists to conduct research addressing fundamental social problems [28]. Given the high cost of fundraising—$1US for every $6 collected [29]—it is critical to understand how to encourage donations among those with the greatest capacity to give. Rather than simply encouraging everyone to work together, our data suggest it help to highlight the unique role that each individual can play.

## Supporting information

S1 Text This contains all supporting information, including Table A, Table B, Table C, and Campaign materials.
(DOCX)
