# Supplementary material for: Agentic appeals increase charitable giving in an affluent sample of donors
Source: PLoS One. 2018 Dec 6;13(12):e0208392. doi: 10.1371/journal.pone.0208392 (PMC6283602; doi:10.1371/journal.pone.0208392)
Supplement: S1 Text — This contains all supporting information, including Table A, Table B, Table C, and Campaign materials. (DOCX) [file pone.0208392.s001.docx]

**Campaign analyses.** Alumni of the participating university received three campaign messages (two emails and one mail-out) over a 30-day campaign cycle. These emails and the mail-out campaign framed donations to the campaign as an agentic or as a communal act.

In the main text, we report the effects collapsing across the fundraising campaigns to maximize the power to detect the effects of condition assignment on the amount that individuals donated. On an exploratory basis, we report the effects of condition on donation amount looking at each campaign separately. It is worth noting that these analyses are underpowered given the low number of donations made to each campaign (vs. collapsing across campaigns).

Among individuals who made a donation to the mail-out campaign (*N* = 375), participants who were assigned to view the agentic appeal donated significantly more to the campaign (*M* = $446.31, *SD* = $400.68) as compared to participants who were assigned to view the communal appeal (*M* = $272.76, *SD* = $857.19), *F*(1, 375) = 6.06, *p* = 0.014, η^2^ = 0.016.

Among individuals who made a donation to the first email campaign (*N* = 118), participants who were assigned to view the agentic appeal donated more to the campaign (*M* = $383.36, *SD* = $766.77) compared to participants who were assigned to view the communal appeal (*M* = $258.25, *SD* = $340.41), *F*(1, 117) = 1.28, *p* = 0.260, η^2^ = 0.011; this result was not statistically significant given the low power of this analysis.

Among individuals who made a donation to the second email campaign (*N* = 12), participants who were assigned to view the agentic appeal donated more (*M* = $548.75, *SD* = $719.63) as compared to participants who were assigned to view the communal appeal (*M* = $162.50, *SD =* $225.00), *F*(1, 11) = 1.05, *p* = 0.33, η^2^ = 0.10; given the extremely low power to detect effects, this result was not statistically significant. These analyses provide additional evidence that the effect of the agentic vs. communal appeals were consistent across the campaigns in which they were implemented.

**Additional Condition Analyses.** In the analyses reported in text, we focus our analyses on OLS regressions predicting raw donation amount as the key outcome of interest. It is worth noting that all of the analyses reported in text also hold when we use a log-transformed donation variable as the key outcome variable of interest and when running tobit analsyes (Table 2&3).

| Predictors | Probit  Model 1 ^a^ | Probit  Model 2^b^ |
| --- | --- | --- |
| Condition (1=Agentic) | 0.057 (0.04) | 0.062  (0.05) |
| Income |  | 0.000001  (0.000002) |
| Gender (1=Female) |  | -0.04  (0.07) |
| Age |  | 0.02**  (0.002) |
| Warm List Last Year (1=Yes) |  | 0.96**  (0.07) |
| Warm List Ever (1=Yes) |  | 0.69**  (0.13) |
| Years of Donations |  | 0.03**  (0.004) |
| Amount of Past Donations |  | -0.0000001  (0.000002) |
| Model Chi-Square | 1.97 | 1,084.69 |
| *Dfs* | 1 | 8 |
| *N* | 12,316 | 12,316 |
| P-value for the model  Pseudo R^2^ | 0.160  0.001 | <0.001  0.093 |

†*p* *< .*10, **p* < .05, ***p* < .01

| Predictors | OLS  Model 1^a^ | OLS  Model 2^b^ | OLS  Model 3^c^ | OLS  Model 4^d^ | OLS  Model 5^e^ | OLS  Model 6^f^ | Tobit Model 7^g^ | Tobit Model 8^h^ |
| --- | --- | --- | --- | --- | --- | --- | --- | --- |
| Condition (1=Agentic) | $8.34**  ($2.73) | $7.88**  ($2.70) | $1.66* ($0.70) | $3.32*  ($1.441) | $7.485** ($2.762) | $0.01* ($0.007) | 100.30* (46.48) | 114.12* (48.82) |
| Income |  | $0.001 ($0.00004) | -$0.0001 ($0.0001) | $0.0001 ($0.0001) | -$0.0001 ($0.001) | $0.0001 ($0.0001) |  | -0.007 (0.0007) |
| Gender (1=Female) |  | -$4.19  ($3.21) | $0.052 ($0.824) | $0.388 ($1.698) | -$0.537 ($3.255) | -$0.02 ($0.009) |  | -150.42** (61.37) |
| Age |  | $0.006  ($0.005) | $0.229** ($0.029) | $0.296** ($0.059) | $0.307** ($0.113) | $0.00005** ($0.00001) |  | 0.29** (0.09) |
| Warm List Last Year (1=Yes) |  | $28.83**  ($3.90) | $8.679** ($0.987) | $14.734** ($2.034) | $23.412**  ($3.898) | $0.11** ($0.01) |  | 965.34** (74.17) |
| Warm List Ever (1=Yes) |  | $2.37  ($3.24) | $3.869** ($0.913) | $4.385* ($1.882) | $4.018 ($3.606) | $0.06** ($0.01) |  | 511.40** (120.68) |
| Years of Donations |  | $3.27**  ($0.36) | $1.976** ($0.097) | $2.840** ($0.200) | $2.347** ($0.383) | $0.03**  ($0.001) |  | 36.51** (3.34) |
| Amount of Past Donations |  | $0.0006 (0.00001) | $0.0001 ($0.0001) | $0.001** ($0.0001) | $0.002**  ($0.0001) | $0.0001  ($0.0001) |  | -0.0002 ($0.00001) |
| Model F-value/Chi-Square | 9.31 | 42.72 | 161.77 | 99.46 | 58.77 | 256.90 | 4.67 | 1,047.83 |
| *Dfs* | 1 | 8 | 8 | 8 | 8 | 8 | 1 | 8 |
| *N* | 12,316 | 12,306 | 12,306 | 12,306 | 12,306 | 12,306 | 12,316 | 12,306 |
| P-value for the model  Adjusted R-square/PseudoR^2^ | 0.002  0.001 | <0.001  0.026 | <0.001  0.10 | <0.001  0.07 | <0.001  0.040 | <0.001  0.144 | 0.0306  0.004 | <0.001  0.094 |

†*p* *< .*10, **p* < .05, ***p* < .01

^a^Model 1 = Condition without covariates predicting raw donation amount.

^b^Model 2 = Condition with covariates predicting raw donation amount.

^c^Model 3 = Condition with covariates predicting donation amount windsorized at 90^th^ percentile.

^d^Model 4 = Condition with covariates predicting donation amount windsorized at 95^th^ percentile.

^e^Model 5 = Condition with covariates predicting donation amount windsorized at 99^th^ percentile.

^f^Model 6 = Condition with covariates predicting donation amount log transformed. To log-transform we added +1 to all 0’s.

^g^Model 7 = Condition without covariates predicting donation amount. Tobit regression analyses to account for 0’s.

^h^Model 7 = Condition with covariates predicting donation amount. Tobit regression analyses to account for 0’s.

| Predictors | OLS  Model 1^a^ | OLS  Model 2^b^ | OLS  Model 3^c^ | OLS  Model 4^d^ | OLS  Model 5^e^ | OLS  Model 6^f^ | Tobit Model 7^h^ | Tobit Model 8th |  |
| --- | --- | --- | --- | --- | --- | --- | --- | --- | --- |
| Condition (1=Agentic) | $7.18** ($2.63) | $7.48** ($2.82) | $1.715* ($0.699) | $3.472* ($1.440) | $8.126** ($2.749) | $0.007* ($0.004) | 88.23*  (54.88) | 135.94** (51.54) |  |
| Past Donations | $29.30** ($1.42) | $24.89** ($1.53) | $0.0001* ($0.0001) | $0.0001 ($0.0001) | $0.0001 ($0.0001) | -$0.003 ($0.004) | 37.81 (27.23) | 69.80 (27.26) |  |
| Condition X Past Donations | $20.39** ($1.42) | $22.33** ($1.48) | $0.0001** ($0.001) | $0.001** ($0.0001) | $0.003** ($0.0001) | $0.020** ($0.004) | 288.96** (39.69) | 99.56** (26.49) |  |
| Income |  | $0.0001 ($0.0001) | $0.0001 ($0.0001) | $0.0001 ($0.001) | $0.0001 ($0.001) | $0.0001 ($0.0001) |  | 0.0001 (0.007) |  |
| Gender (1=Female) |  | -$0.52 ($3.32) | $0.057 ($0.824) | $0.403 ($1.697) | -$0.473 ($3.239) | -$0.003 ($0.009) |  | -37.17 (67.42) |  |
| Age |  | $0.27* ($0.12) | $0.229** ($0.029) | $0.295** ($0.059) | $0.303** ($0.113) | $0.003** ($0.0001) |  | 0.003 (0.09) |  |
| Warm List Last Year (1=Yes) |  | $24.61** ($3.97) | $8.680** ($0.987) | $14.738** ($2.032) | $23.427** ($3.879) | $0.11** ($0.011) |  | 125.55 (93.85) |  |
| Warm List Ever (1=Yes) |  | $2.90 ($3.67) | $3.872** ($0.913) | $4.391** ($1.880) | $4.045** ($3.589) | $0.06** ($0.010) |  | 150.18 (205.46) |  |
| Years of Donations |  | $2.16** ($0.39) | $1.980** ($0.097) | $2.851** ($0.200) | $2.395** ($0.382) | $0.03** ($0.001) |  | 0.01 (0.002)** |  |
| F-value/Chi-Square | 336.44 | 127.49 | 145.39 | 91.07 | 64.59 | 204.00 | 106.14 | 177.94 |  |
| *Dfs* | 3 | 9 | 9 | 9 | 9 | 9 | 3 | 9 |  |
| *N* | 12,316 | 11,079 | 11,070 | 11,070 | 11,070 | 11,079 | 12,316 | 12,316 |  |
| P-value for the model  R-square/Pseudo R^2^ | < 0.001  0.08 | <0.001  0.09 | <0.001  0.11 | <0.001  0.07 | <0.001  0.05 | <0.001  0.14 | <0.001  0.014 | <0.001  0.022 |  |

†*p* *< .*10, **p* < .05, ***p* < .01

^a^Model 1 = Condition without covariates predicting raw donation amount.

^b^Model 2 = Condition with covariates predicting raw donation amount.

^c^Model 3 = Condition with covariates predicting donation amount windsorized at 90^th^ percentile.

^d^Model 4 = Condition with covariates predicting donation amount windsorized at 95^th^ percentile.

^e^Model 5 = Condition with covariates predicting donation amount windsorized at 99^th^ percentile.

^f^Model 6 = Condition with covariates predicting donation amount log transformed. To log-transform we added +1 to all 0’s.

^g^Model 7 = Condition without covariates predicting donation amount. Tobit regression analyses to account for 0’s.

^h^Model 8 = Condition with covariates predicting donation amount. Tobit regression analyses to account for 0’s.

| Condition  **Communal**  December, 2015  Dear <<SALUTATION>>,  Sometimes, one community needs to come forward and support a common goal. This is one of those times.  [Institution Name] is a business school with few peers. The [Institution Name] approach to business education has shaped the school so that it is among the elite few where research and ideas are paramount. It is a place that produces first rate ideas and educates leaders who create real change.  [Institution Leader’s] vision for the campaign is to build upon the school’s two most significant advantages. One strength lies in its flexibility—the freedom to enroll in any combination of courses or to take risks in research and experimentation. Another benefit is its academically rigorous culture which has produced incredible thought leaders among the faculty and students who are drawn to solving problems and challenging conventional wisdom.  You are an important part of [Institution Name] and have contributed to its success. Thank you.  This campaign seeks to invest in the very people that embody the business school—its students, faculty, and alumni.  If you are planning to give to the school, now is the time to make an impact. Please consider a gift of <<SPECIFIC ASK>>.  Join your community and support a common goal. Donate today.  With appreciation,  Director of Annual Funds  P.S. Learn more and make your gift at [[Link to website]](http://Campaign.ChicagoBooth.edu/) |
| --- |
|  |
| **Agentic**  December, 2015  Dear <<SALUTATION>>,  Sometimes, one person needs to come forward and take individual action. This is one of those times.  [Institution Name] is a business school with few peers. The [Institution Name] approach to business education has shaped the school so that it is among the elite few where research and ideas are paramount. It is a place that produces first rate ideas and educates leaders who create real change.  [Institution Leader’s] vision for the campaign is to build upon the school’s two most significant advantages. One strength lies in its flexibility—the freedom to enroll in any combination of courses or to take risks in research and experimentation. Another benefit is its academically rigorous culture which has produced incredible thought leaders among the faculty and students who are drawn to solving problems and challenging conventional wisdom.  You are an important part of [Institution Name] and have contributed to its success. Thank you.  This campaign seeks to invest in the very people that embody the business school—its students, faculty, and alumni.  If you are planning to give to the school, now is the time to make an impact. Please consider a gift of <<SPECIFIC ASK>>.  Come forward and take individual action. Donate today.  With appreciation,  Director of Annual Funds  P.S. Learn more and make your gift at [[Link to website]](http://Campaign.ChicagoBooth.edu/) |

*Note.* The name of the university and small detailed are changed to maintain the confidentiality of the partner university.
